# TSH Stimulation Reveals Additional Bone Metastases on [^18^F]FDG PET/CT in Metastatic Follicular Thyroid Carcinoma

**DOI:** 10.3390/diagnostics16193146

**Published:** 2026-09-28

**Authors:** Jang Bae Moon, Sang-Geon Cho, Sae-Ryung Kang, Jahae Kim, Seong-Young Kwon, Su Woong Yoo

**Affiliations:** 1 Departments of Nuclear Medicine, Chonnam National University Medical School and Hwasun Hospital, Hwasun-gun 58128, Republic of Korea; 2 Departments of Nuclear Medicine, Chonnam National University Medical School and Hospital, Gwangju 61469, Republic of Korea

**Keywords:** [^18^F]FDG PET/CT, recombinant human TSH, follicular thyroid carcinoma, bone metastasis, differentiated thyroid cancer, molecular imaging

## Abstract

A 62-year-old woman with metastatic follicular thyroid carcinoma underwent 2-deoxy-2-[^18^F]fluoro-D-glucose ([^18^F]FDG) PET/CT before a second course of radioactive iodine therapy because of a markedly elevated serum thyroglobulin level (498.0 ng/mL). The initial PET/CT was performed under thyroid-stimulating hormone (TSH) suppression (0.01 μIU/mL) and demonstrated FDG uptake in known osseous metastases involving the T10 and L5 vertebrae, while additional skeletal metastases were not conspicuous. Repeat PET/CT performed 6 days later after recombinant human TSH (rhTSH) stimulation (TSH, 70.96 μIU/mL) showed substantially increased FDG uptake in the known T10 and L5 lesions, with SUVmax increasing from 3.33 to 6.31 and from 3.98 to 11.61, respectively. In addition, metastases in the T9 vertebra and sternum became conspicuous only after TSH stimulation and remained evident on subsequent follow-up imaging, supporting their metastatic nature. This paired within-patient comparison directly demonstrates that, in this patient, osseous lesions inconspicuous under TSH suppression became clearly visualized after rhTSH stimulation and persisted on follow-up imaging, illustrating the potential influence of TSH status on the apparent skeletal metastatic burden on [^18^F]FDG PET/CT.

**Figure 1:**
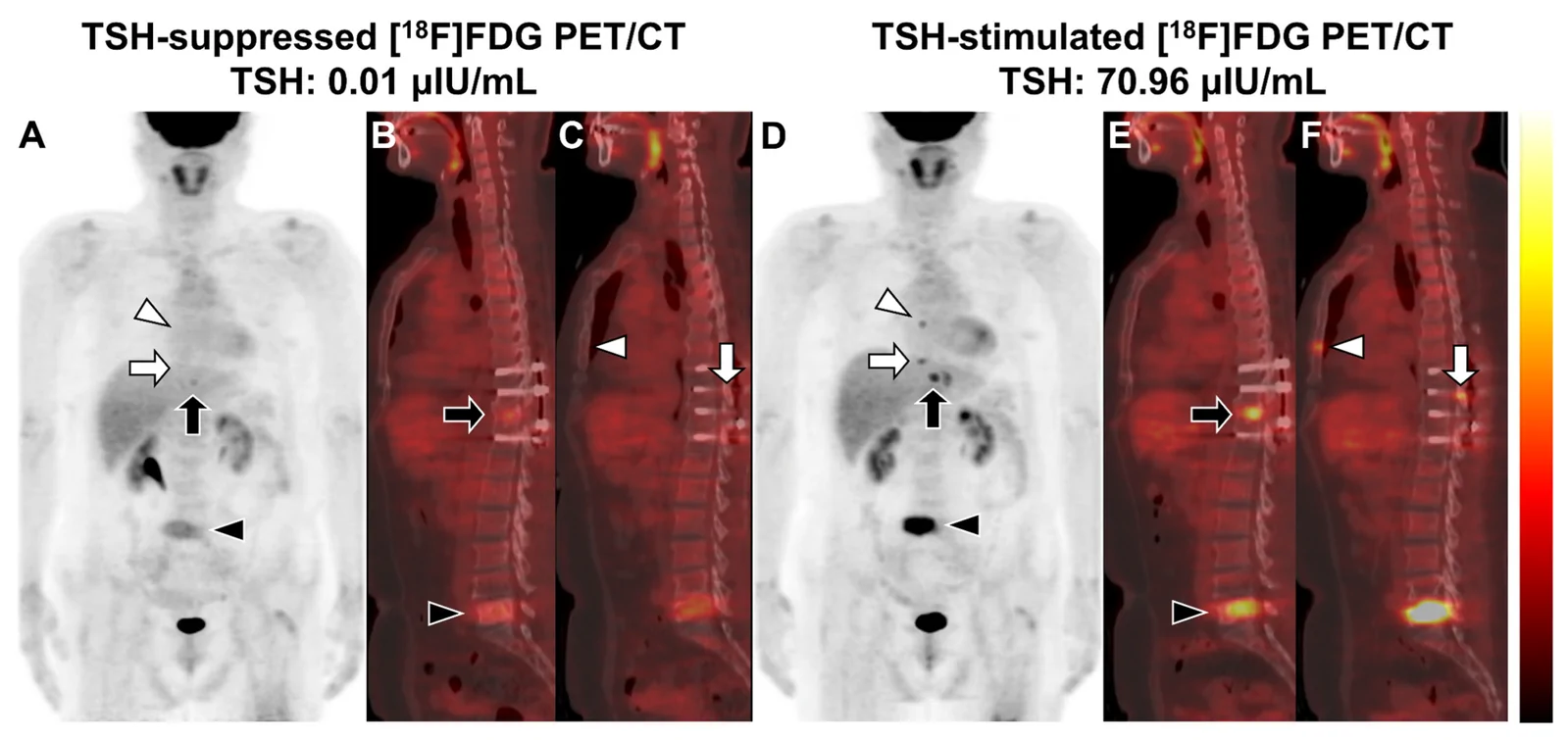
TSH stimulation increases the conspicuity of known osseous metastases and reveals additional bone metastases on [^18^F]FDG PET/CT in follicular thyroid carcinoma. The color scale represents the relative intensity of [^18^F]FDG uptake. A 62-year-old woman with metastatic follicular thyroid carcinoma (FTC) underwent T10 laminectomy with tumor removal for a metastatic vertebral lesion, followed by total thyroidectomy with selective neck dissection and palliative radiotherapy to the T10 and L5 vertebral metastases. After the first radioactive iodine (RAI) therapy with 7.4 GBq (200 mCi) of ^131^I, a left femoral shaft metastasis complicated by a pathologic fracture was treated with intramedullary nailing and palliative radiotherapy. Before a second course of RAI therapy, her serum thyroglobulin level remained markedly elevated at 498.0 ng/mL, prompting 2-deoxy-2-[^18^F]fluoro-D-glucose ([^18^F]FDG) PET/CT to evaluate the extent of metastatic disease. The initial [^18^F]FDG PET/CT was performed under TSH suppression (serum TSH, 0.01 μIU/mL) and demonstrated FDG uptake in the previously known T10 and L5 vertebral metastases (**A**–**C**). Given the multifocal distribution of osseous metastatic disease, including involvement of the axial skeleton and left femoral shaft, a more comprehensive assessment of the overall skeletal metastatic burden was clinically warranted. Therefore, repeat [^18^F]FDG PET/CT was performed 6 days later, after two consecutive daily intramuscular injections of recombinant human TSH (rhTSH, 0.9 mg; Thyrogen, Sanofi Genzyme, Cambridge, MA, USA) administered 24 h apart; the stimulated study was acquired approximately 24 h after the second injection. At the time of the second examination, the serum TSH level had increased to 70.96 μIU/mL (**D**–**F**). Both examinations were performed on the same PET/CT scanner (Discovery IQ; GE Healthcare, Milwaukee, WI, USA) using identical acquisition and reconstruction protocols (2 min per bed position; ordered-subset expectation maximization with point-spread-function modeling [VUE Point HD with SharpIR], 2 iterations, 12 subsets, 192 × 192 matrix), with comparable administered [^18^F]FDG activity (151.7 vs. 150.6 MBq) and uptake time (approximately 60 min). The pre-injection blood glucose levels were 99 and 116 mg/dL for the TSH-suppressed and rhTSH-stimulated examinations, respectively. SUVmax was measured by the same reader using a manually placed volume of interest encompassing the entire lesion on fused PET/CT images, and was defined as the maximum voxel value within the volume of interest; corresponding lesions were evaluated at the same anatomical levels on both examinations. The rhTSH-stimulated study demonstrated a marked increase in FDG uptake in the known osseous metastases. The SUVmax of the T10 vertebral metastasis (black arrow) increased from 3.33 under TSH suppression to 6.31 after TSH stimulation, whereas that of the L5 vertebral metastasis (black arrowhead) increased from 3.98 to 11.61. More importantly, additional metastases involving the T9 vertebra (white arrow) and sternum (white arrowhead), which were not conspicuous on the TSH-suppressed study, became clearly visible after rhTSH stimulation. On subsequent follow-up imaging, the sternal lesion showed a corresponding osteolytic abnormality on CT performed approximately 4 months later, while CT evaluation of the T9 lesion was limited by metallic artifacts; both lesions remained metabolically evident on [^18^F]FDG PET/CT performed approximately 1 year later, further supporting their metastatic nature. The patient subsequently underwent a second RAI treatment with 7.4 GBq (200 mCi) of ^131^I on the day following the stimulated PET/CT. Because multifocal osseous metastatic disease had already been established, the identification of the additional T9 and sternal metastases did not alter the immediate therapeutic plan, including the previously scheduled second RAI treatment; rather, their detection provided a more complete delineation of the overall skeletal metastatic burden and a baseline for subsequent imaging follow-up. FTC has a greater propensity for hematogenous dissemination than papillary thyroid carcinoma, with the lungs and skeleton representing important sites of distant metastatic disease [1,2]. Bone metastases are clinically relevant because they can cause pain, pathological fractures, neurologic complications, and other skeletal-related events and may substantially affect therapeutic planning and prognosis [3]. Although conventional anatomical imaging and radioiodine whole-body imaging remain important in the evaluation of differentiated thyroid carcinoma, [^18^F]FDG PET/CT provides complementary metabolic information and is particularly useful in patients with biochemical or clinical evidence of persistent or recurrent disease when conventional or radioiodine imaging does not fully characterize the disease burden [4,5]. The biological basis for increased FDG uptake after TSH stimulation is supported by experimental observations demonstrating that TSH can stimulate glucose transport and glucose metabolism in thyroid cells [6,7]. This provides a plausible mechanism by which rhTSH stimulation may increase the metabolic conspicuity of differentiated thyroid cancer lesions on [^18^F]FDG PET/CT. Clinical investigations have also evaluated the incremental value of rhTSH-stimulated [^18^F]FDG PET/CT. Leboulleux et al. reported improved lesion localization in a subset of patients following recombinant thyrotropin stimulation, while Rendl et al. demonstrated that pre-ablation rhTSH-stimulated [^18^F]FDG PET/CT could provide additional information with an impact on patient management in increased-risk thyroid cancer [8,9]. However, the incremental diagnostic and clinical value of TSH stimulation before [^18^F]FDG PET/CT remains debated [10,11]: in a recent retrospective analysis, rhTSH stimulation increased the number of visualized lesions but did not improve the overall rate of positive PET/CT results [10]. In this context, the present case provides a direct within-patient imaging comparison demonstrating two potentially relevant effects of TSH stimulation**:** increased metabolic conspicuity of already recognized osseous metastases and visualization of additional metastatic lesions that were inconspicuous under TSH suppression. Importantly, the persistence of the additionally detected T9 and sternal lesions on subsequent imaging follow-up supports that these findings represented true metastatic disease rather than transient or nonspecific FDG uptake. Nevertheless, because multifocal osseous metastatic disease had already been established in this patient, the detection of the additional lesions did not change the overall therapeutic intent, and in advanced metastatic disease the identification of further bone lesions may have limited influence on management. The principal teaching point of this case is therefore that TSH status at the time of imaging may influence both the metabolic conspicuity and the apparent extent of osseous metastatic disease on [^18^F]FDG PET/CT. Current American Thyroid Association guidelines recognize either rhTSH-mediated TSH stimulation or thyroid hormone withdrawal as preparation options for RAI therapy in patients with distant metastatic differentiated thyroid carcinoma [12]. In the present case, rhTSH was administered as preparation for the planned second RAI treatment rather than specifically to enhance [^18^F]FDG PET/CT. As this represents a single opportunistic observation without a control group, these findings cannot be generalized. Whether such improved lesion detection could translate into clinically meaningful changes in staging or management remains to be determined, and prospective studies would be required before rhTSH-stimulated [^18^F]FDG PET/CT could be recommended for routine clinical use.

## Data Availability

The data underlying this article are not publicly available because they contain potentially identifiable clinical information and medical images. De-identified data may be made available from the corresponding author upon reasonable request and with appropriate institutional approval.
